# Osteosarcopenia: More Than the Sum of Its Parts? A Community-Based Study Across Sarcopenia Definitions

**DOI:** 10.1007/s00223-026-01611-w

**Published:** 2026-10-01

**Authors:** Radmila Matijevic, Milan Tosic, Filip Lazetic, Nikola Vukosav, Milan Majkic, Branko Baljak, Dora Selesi

**Affiliations:** University of Novi Sad Faculty of Medicine, Novi Sad, Vojvodina Serbia

**Keywords:** Osteoporosis, Sarcopenia, Osteosarcopenia, Prevalence, Hip structural analysis, Muscle-bone unit

## Abstract

Osteosarcopenia, the co-existence of osteoporosis and sarcopenia, is described as a geriatric syndrome arising from shared determinants of muscle and bone. We asked how far the prevalence of sarcopenia and osteosarcopenia depends on the definition of low muscle mass, whether osteoporosis and sarcopenia co-occur more often than expected under statistical independence, and whether the muscle–bone relationship is expressed in areal bone mineral density (BMD) or in femoral geometry. Cross-sectional study of 649 community-dwelling adults aged 65 years and over (72% women; mean age 71.5 years; mean body mass index (BMI) 29.6 kg/m^2^) assessed with dual-energy X-ray absorptiometry (GE Lunar iDXA), grip dynamometry (Jamar Hydraulic Hand Dynamometer) and the Short Physical Performance Battery. Osteoporosis followed World Health Organization criteria. Sarcopenia was classified according to EWGSOP2, and low muscle mass was defined three ways (appendicular lean mass (ALM)/height2; absolute ALM; and the Foundation for the National Institutes of Health (FNIH) ALM/BMI index). Observed co-occurrence was compared with the value expected under independence (Fisher exact test, odds ratios (OR) with 95% confidence intervals (CI)); for the best-powered analysis the two one-sided tests (TOST) equivalence procedure using pragmatic equivalence bounds of OR 0.5 to 2.0. Hip structural analysis provided section modulus and cross-sectional area. Low muscle mass ranged from 3.7 to 15.7%, and confirmed sarcopenia from 1.4 to 4.2%, according to the metric. Osteoporosis prevalence was 17.6% (95% CI 14.9 to 20.8). Under none of the sarcopenia definitions was there statistically significant evidence of excess co-occurrence (Fisher *p* = 0.50 to 1.00), although observed overlap under the absolute-ALM definition was slightly above the independence expectation. For probable sarcopenia (low grip strength), the best-powered comparison, the OR was 0.89 (95% CI 0.47 to 1.67) and equivalence within broad OR bounds of 0.5 to 2.0 was supported (90% CI 0.52 to 1.51; TOST *p* = 0.037); equivalence was not supported under narrower bounds. Appendicular lean mass was not associated with areal BMD after adjustment for body size (femoral neck standardised β = 0.03, 95% CI − 0.18 to 0.24) but was associated with femoral section modulus substantially more strongly in men than women (globally standardised interaction Δβ = 0.30, 95% CI 0.15 to 0.44, *p* < 0.001). In this community sample, there was no statistically significant evidence that osteoporosis and sarcopenia co-occurred more often than expected under statistical independence, across several sarcopenia definitions, although estimates for confirmed sarcopenia were imprecise. The muscle-bone association was more apparent for femoral geometry than areal BMD and was seen predominantly in men. These findings do not support assuming that low bone mass and low muscle mass identify the same individuals, but they concern statistical co-occurrence and do not establish biological independence.

**Supplementary Information:**

The online version contains supplementary material available at https://doi.org/10.1007/s00223-026-01611-w.

## Introduction

Osteoporosis and sarcopenia are common disorders of older age. Osteoporosis is defined densitometrically by a bone mineral density (BMD) T-score at or below −2.5 [1, 2]; sarcopenia by the revised European consensus (EWGSOP2) based on low muscle strength, low muscle quantity and, in severe cases, poor physical performance [3]. Their coexistence, termed osteosarcopenia, was proposed as a distinct geriatric syndrome arising from shared genetic, endocrine, mechanical, and lifestyle determinants of muscle and bone [4, 5], and is reported to carry an increased risk of falls, fractures, and mortality [6–9].

This framing carries an implicit assumption: namely, that low bone mass and low muscle mass co-segregate in the same individuals more often than chance would predict. Whether this holds in the general older population, rather than in selected clinical samples, is uncertain, and the question of whether osteoporosis and sarcopenia are “two diseases or one” remains open [10, 11]. The issue is complicated because the prevalence of sarcopenia, and hence of osteosarcopenia, depends heavily on how low muscle mass is operationalised, particularly in overweight populations where height-squared normalisation and BMI adjustment behave very differently [3, 12, 13].

There is also a structural reason to examine the assumption. Muscle and bone are functionally coupled, and the mechanostat model holds that muscle loading shapes the strength-relevant geometry of bone [14, 15]. Areal BMD, however, is a size-dependent two-dimensional projection that does not directly capture bone geometry, whereas hip structural analysis derives geometric indices of strength from the same image [16, 17]. If the muscle–bone relationship is expressed mainly in geometry rather than in areal BMD, the two axes on which osteosarcopenia is defined, the T-score and DXA muscle indices, may be only weakly related, which would be compatible with a low co-occurrence.

We therefore examined a community-dwelling cohort of older adults, with the aims of (1) describing the prevalence of osteoporosis, sarcopenia and osteosarcopenia and its dependence on the definition of low muscle mass; (2) testing whether osteoporosis and sarcopenia co-occur more often than expected under statistical independence; and (3) exploring whether the muscle–bone relationship is expressed in areal BMD or in femoral geometry.

## Materials and Methods

### Study Design and Participants

**Fig. 1:**
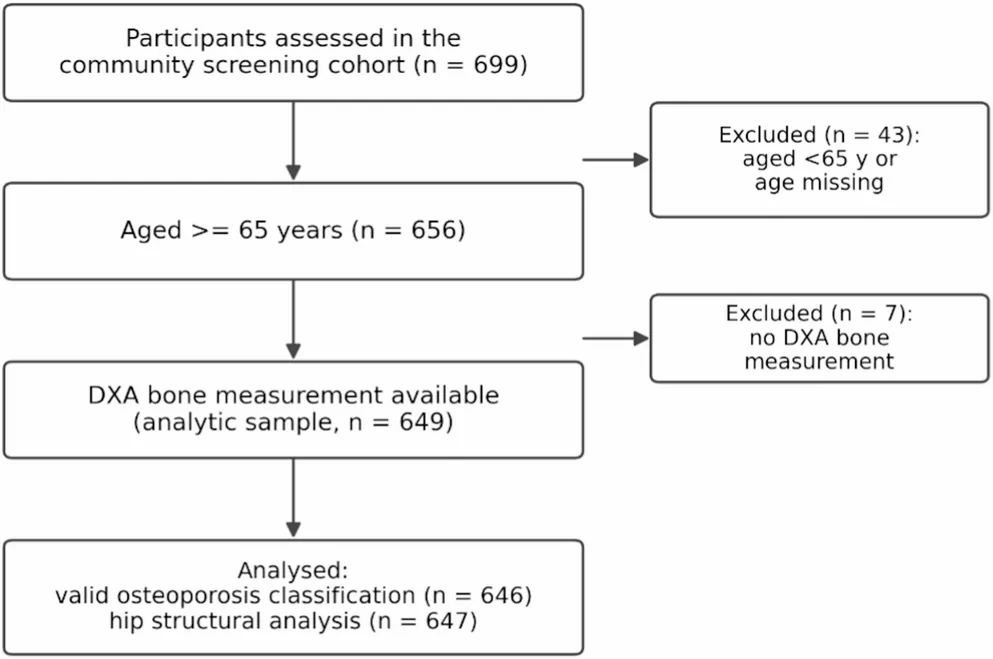
Flow of participants through the study

This cross-sectional analysis was based on a community screening study of older adults in the Vojvodina region of Serbia, conducted at the University Clinical Centre of Vojvodina in Novi Sad. Participants were community-dwelling and aged 65 years and over. Reporting follows the STROBE guidance for cross-sectional studies. Of 699 participants assessed, 43 were excluded (42 aged under 65 years, one with missing age), and 7 had no dual-energy X-ray absorptiometry (DXA) bone measurements, leaving an analytic sample of 649, of whom 646 had a valid osteoporosis classification, and 647 had a hip structural analysis (Fig. 1). Ethical approval was granted by the University Clinical Centre of Vojvodina Ethics Committee, approval number 6 00–65/2, and all participants gave written informed consent.

### Bone and Body Composition

Whole-body, lumbar spine (L1–L4) and proximal femur scans were acquired on a GE Lunar iDXA densitometer using the manufacturer's standard acquisition and positioning protocols, with daily quality control against the manufacturer's phantom. T-scores were derived from the manufacturer reference database. Appendicular lean mass (ALM) was the sum of lean mass in the four limbs. Hip structural analysis was performed on the left hip at the narrow femoral neck region and was available for 647 of the 649 participants in the present analytic cohort, yielding the section modulus (an index of resistance to bending) and cross-sectional area; scans with an implausible buckling ratio (> 30, n = 5) were examined in sensitivity analyses. Site-specific short-term precision (coefficient of variation) for the geometric indices was not available and is acknowledged as a limitation.

### Muscle Strength and Physical Performance

Grip strength was measured by hand dynamometry (Jamar Hydraulic Hand Dynamometer—maximum of the recorded values). Physical performance was assessed with the Short Physical Performance Battery (SPPB), reconstructed to the standard 0 to 12 scale from raw balance, 4 m gait time and five-repetition chair-rise time; the reconstructed total agreed with a previously recorded total (r = 0.94; 96.9% within 1 point) [18].

### Definitions

Osteoporosis was the lowest T-score of the femoral neck, total hip or L1–L4 at or below −2.5, with osteopenia between −2.5 and −1.0 [1, 2]. Sarcopenia was assessed using the EWGSOP2 criteria [3]: probable sarcopenia was defined as low grip strength (< 27 kg in men, < 16 kg in women); confirmed sarcopenia was defined as low grip strength with low muscle mass. Low muscle mass was operationalised three ways: ALM/height^2^ (< 7.0 kg/m^2^ men, < 5.5 kg/m^2^ women), the EWGSOP2 reference metric [3]; absolute (non-normalised) ALM (< 20 kg men, < 15 kg women), also specified by EWGSOP2 [3]; and the FNIH ALM/BMI index (< 0.789 men, < 0.512 women), used as the primary metric given the high mean BMI of the cohort [12, 13]. Osteosarcopenia was osteoporosis with confirmed sarcopenia.

### Statistical Analysis

Analyses were performed in Python with standard libraries (numpy, pandas, matplotlib) using a single de-identified patient dataset; all analyses were complete-case for the variables required in the relevant table or model, and the available sample size is reported for each analysis. Prevalence is reported with exact ratios and 95% CIs from Wilson. To assess co-occurrence, the observed prevalence of osteosarcopenia was compared descriptively with the value expected under statistical independence (the product of the two marginal prevalences); the formal test of association was the Fisher exact test, with the odds ratio (OR) and 95% CI. Co-occurrence analyses were repeated by sex and by osteoporosis site (femoral neck, total hip, hip-only, lumbar spine and any-site definitions) as sensitivity analyses. Because absence of a significant association does not establish independence, we additionally applied, for the best-powered analysis (probable sarcopenia, i.e. low grip strength), a two-one-sided tests (TOST) equivalence procedure using pragmatic equivalence bounds of OR 0.5 to 2.0 (deliberately broad), with additional sensitivity bounds of 0.67 to 1.50 and 0.75 to 1.33. Severe sarcopenia was defined as confirmed sarcopenia with SPPB <= 8. Variables associated with prevalent osteoporosis were examined by logistic regression, including crude and adjusted models for each sarcopenia definition.

The definition-dependence of prevalence and the test of co-occurrence against chance were the primary analyses; the muscle–bone regressions, the sex interaction and the within-subject contrast were secondary and exploratory, and the sex-specific findings are presented as requiring external replication. In the secondary analysis, linear regression related appendicular lean mass (ALM) to femoral neck and lumbar spine areal BMD, section modulus, and cross-sectional area, with progressive adjustment for age, sex, BMI, and height, and a sex interaction term. The ALM × sex interaction was estimated with ALM and each outcome standardised across the whole sample, so that the interaction coefficient (Δβ) is interpretable on a common scale; the sex-specific standardised β values in Table 5 were standardised within each sex and are therefore not a simple difference of the interaction term (the unstandardised interaction coefficient is given in Supplementary Table S7). Sensitivity analyses substituted weight for BMI and used an allometric (log–log) specification; influence was assessed using Cook’s distance, leverage, and heteroscedasticity-robust (HC3) standard errors (Supplementary Table S8). Progressive ALM–bone models are shown in Supplementary Table S6. Because lean mass and each bone outcome were measured in the same individuals, the difference between the association of lean mass with section modulus and its association with femoral neck areal BMD was tested within-subject: both outcomes were standardised and their per-participant difference was regressed on lean mass and covariates, with a 95% CI from bootstrap resampling (2000 replicates and a fixed random seed of 42; Supplementary Table S9). Analyses were complete case, with the sample size stated for each. Two-sided *p* < 0.05 was considered significant.

## Results

### Participant Characteristics

The analytic sample comprised 649 older adults (466 women, 183 men) with a mean age of 71.5 years and a mean BMI of 29.6 kg/m^2^ (Fig. 1, Table 1).

**Table 1:** Participant characteristics, overall and by sex (mean ± SD unless stated)

Variable	All	Women	Men
Age, years	71.5 ± 5.2	71.2 ± 4.9	72.3 ± 5.7
Body mass index, kg/m^2^	29.6 ± 4.7	29.9 ± 4.9	28.8 ± 4.0
Height, cm	161.7 ± 8.7	158.1 ± 6.3	170.8 ± 7.3
Appendicular lean mass, kg	19.7 ± 4.2	17.8 ± 2.7	24.5 ± 3.6
ALM/height^2^, kg/m^2^	7.5 ± 1.1	7.1 ± 0.9	8.4 ± 1.0
ALM/BMI	0.67 ± 0.13	0.60 ± 0.09	0.86 ± 0.13
Grip strength, kg	25.8 ± 9.6	21.5 ± 5.9	36.7 ± 8.1
SPPB, median (IQR)	9 (8–11)	9 (8–11)	10 (8–11)
Femoral neck BMD, g/cm^2^	0.84 ± 0.14	0.82 ± 0.13	0.91 ± 0.15
L1–L4 BMD, g/cm^2^	1.15 ± 0.20	1.11 ± 0.19	1.27 ± 0.20
Section modulus, mm^3^	629 ± 218	535 ± 125	868 ± 219
Cross-sectional area, mm^2^	140 ± 31	128 ± 22	170 ± 32
25(OH)D, nmol/L	50.2 ± 20.0	48.7 ± 20.5	53.8 ± 18.1
PTH, ng/L, median (IQR)	71 (52–100)	–	–

### Prevalence Depends Strongly on the Definition of Low Muscle Mass

Osteoporosis was present in 17.6% of participants and rose with age (Table 2). The prevalence of low muscle mass varied more than fourfold across metrics, from 3.7% (ALM/height^2^) to 15.7% (FNIH ALM/BMI), and confirmed sarcopenia ranged from 1.4 to 4.2%. Osteosarcopenia was rare across all definitions (0.2% to 0.6% for confirmed sarcopenia).

**Table 2:** Prevalence of osteoporosis, sarcopenia and osteosarcopenia (Wilson 95% CI)

Category	n/N	% (95% CI)
Osteoporosis (WHO, lowest site ≤ −2.5)	114/646	17.6 (14.9–20.8)
Osteopenia (− 2.5, − 1.0]	316/646	48.9 (45.1–52.8)
Normal (> − 1.0)	216/646	33.4 (29.9–37.2)
Low mass—ALM/height2	24/655	3.7 (2.5–5.4)
Low mass—absolute ALM	81/655	12.4 (10.1–15.1)
Low mass—FNIH ALM/BMI	103/655	15.7 (13.1–18.7)
Probable sarcopenia (low grip)	83/637	13.0 (10.6–15.9)
Confirmed sarcopenia—ALM/height2	9/636	1.4 (0.7–2.7)
Confirmed sarcopenia—absolute ALM	17/636	2.7 (1.7–4.2)
Confirmed sarcopenia—FNIH	27/636	4.2 (2.9–6.1)
Osteosarcopenia (ALM/height2)	1/627	0.2 (0.0–0.9)
Osteosarcopenia (absolute ALM)	4/627	0.6 (0.2–1.6)
Osteosarcopenia (FNIH)	3/627	0.5 (0.2–1.4)

### No Excess Co-occurrence of Osteoporosis and Sarcopenia

**Fig. 2:**
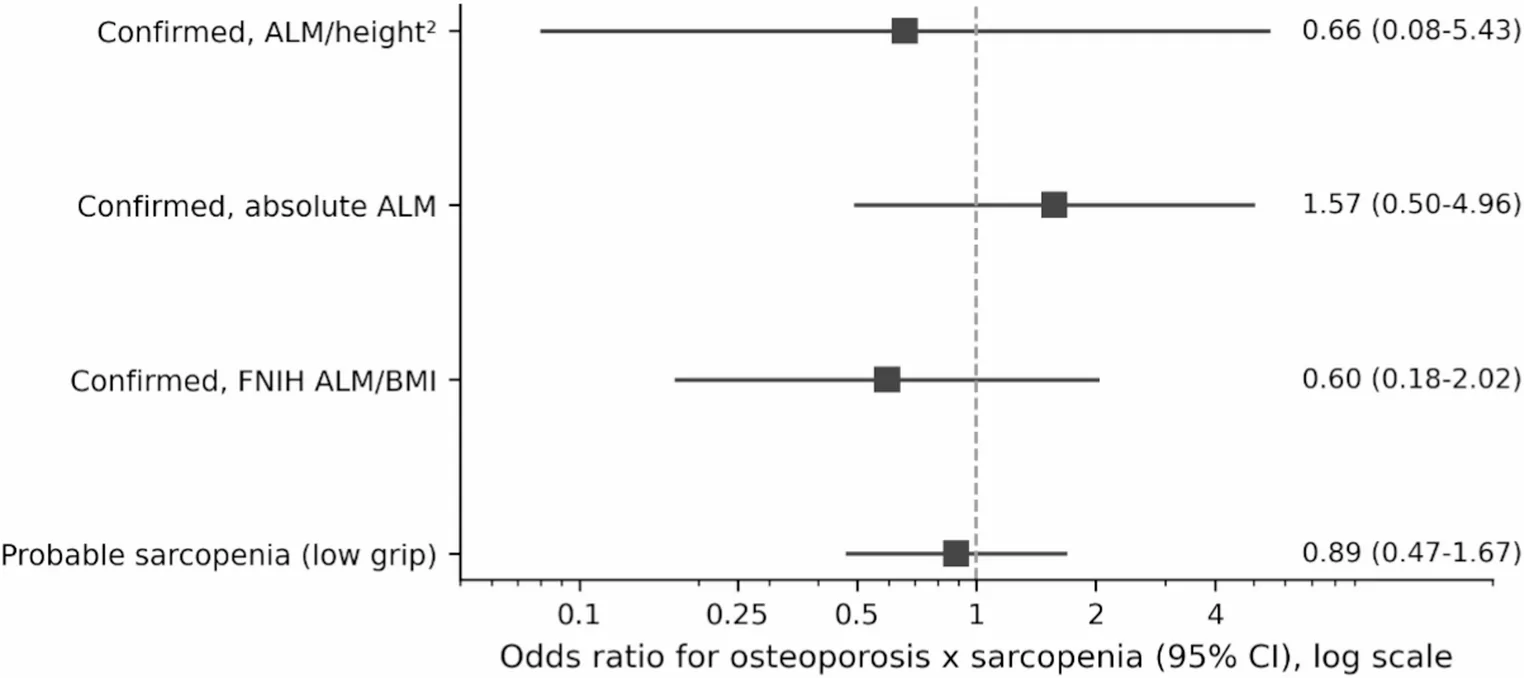
Odds ratios (95% CI) for the association between osteoporosis and sarcopenia under four sarcopenia definitions. All confidence intervals include 1; therefore, no statistically significant departure from independence was detected. The intervals for the confirmed definitions are wide, reflecting few co-occurring cases

Under none of the sarcopenia definitions was there statistically significant evidence of excess co-occurrence (Table 3, Fig. 2). The observed overlap was below the value expected under independence for ALM/height2, FNIH and probable sarcopenia, and slightly above expected for the absolute-ALM definition; the absolute difference was small and the association was not statistically significant. The number of co-occurring cases was small, so the confirmed definitions were imprecise; the raw counts are given in Table 3. Probable sarcopenia provided the largest number of co-occurring cases (13 of 627) and the most precise estimate: the OR for the osteoporosis by sarcopenia association was 0.89 (95% CI 0.47 to 1.67), and equivalence within broad OR bounds of 0.5 to 2.0 was supported (90% CI 0.52 to 1.51; TOST *p* = 0.037). This equivalence conclusion applies only to probable sarcopenia and was not retained under narrower equivalence bounds (Supplementary Table S3). Sex-stratified and osteoporosis-site sensitivity analyses are shown in Supplementary Tables S2 and S4. For the confirmed-sarcopenia definitions, the confidence intervals were wide and compatible with both appreciable positive and negative associations, so the study lacked the precision to assess equivalence. Taken together, these analyses show no evidence of excess co-occurrence rather than positive proof of independence.

**Table 3:** Osteoporosis prevalence by sex and age group (Wilson 95% CI)

Subgroup	n/N	% (95% CI)
Women	95/463	20.5 (17.1–24.4)
Men	19/183	10.4 (6.7–15.6)
65–69	38/269	14.1 (10.5–18.8)
70–79	60/317	18.9 (15.0–23.6)
80+	16/60	26.7 (17.1–39.0)

### Variables Associated with Osteoporosis

Prevalent osteoporosis was associated with older age, female sex and lower BMI (Table 4). When added to the model, confirmed sarcopenia under the FNIH definition was not independently associated with osteoporosis (OR 0.53, 95% CI 0.15 to 1.90). Crude and adjusted models for all sarcopenia definitions showed no statistically significant association with osteoporosis (Supplementary Table S5). Neither 25-hydroxyvitamin D nor PTH was associated with osteoporosis when added to the model.

**Table 4:** Co-occurrence of osteoporosis and sarcopenia versus chance expectation, with raw 2×2 counts, by sarcopenia definition

Sarcopenia definition	OP+/Sarc+; OP+/Sarc-; OP-/Sarc+; OP-/Sarc-	Observed vs expected, %	OR (95% CI)	Fisher p
Confirmed sarcopenia, ALM/height2	1; 110; 7; 509	0.16 vs 0.23	0.66 (0.08–5.43)	1.00
Confirmed sarcopenia, absolute ALM	4; 107; 12; 504	0.64 vs 0.45	1.57 (0.50–4.96)	0.50
Confirmed sarcopenia, FNIH ALM/BMI	3; 108; 23; 493	0.48 vs 0.73	0.60 (0.18–2.02)	0.60
Probable sarcopenia (low grip)	13; 98; 67; 449	2.07 vs 2.26	0.89 (0.47–1.67)	0.88

OP, osteoporosis; Sarc, sarcopenia. Observed vs expected compares the observed osteosarcopenia prevalence with the product of the two marginal prevalences under independence (a descriptive null expectation); the Fisher exact test is the formal test of association

### The Muscle–Bone Relationship is Expressed in Femoral Geometry, More Strongly in Men

**Fig. 3:**
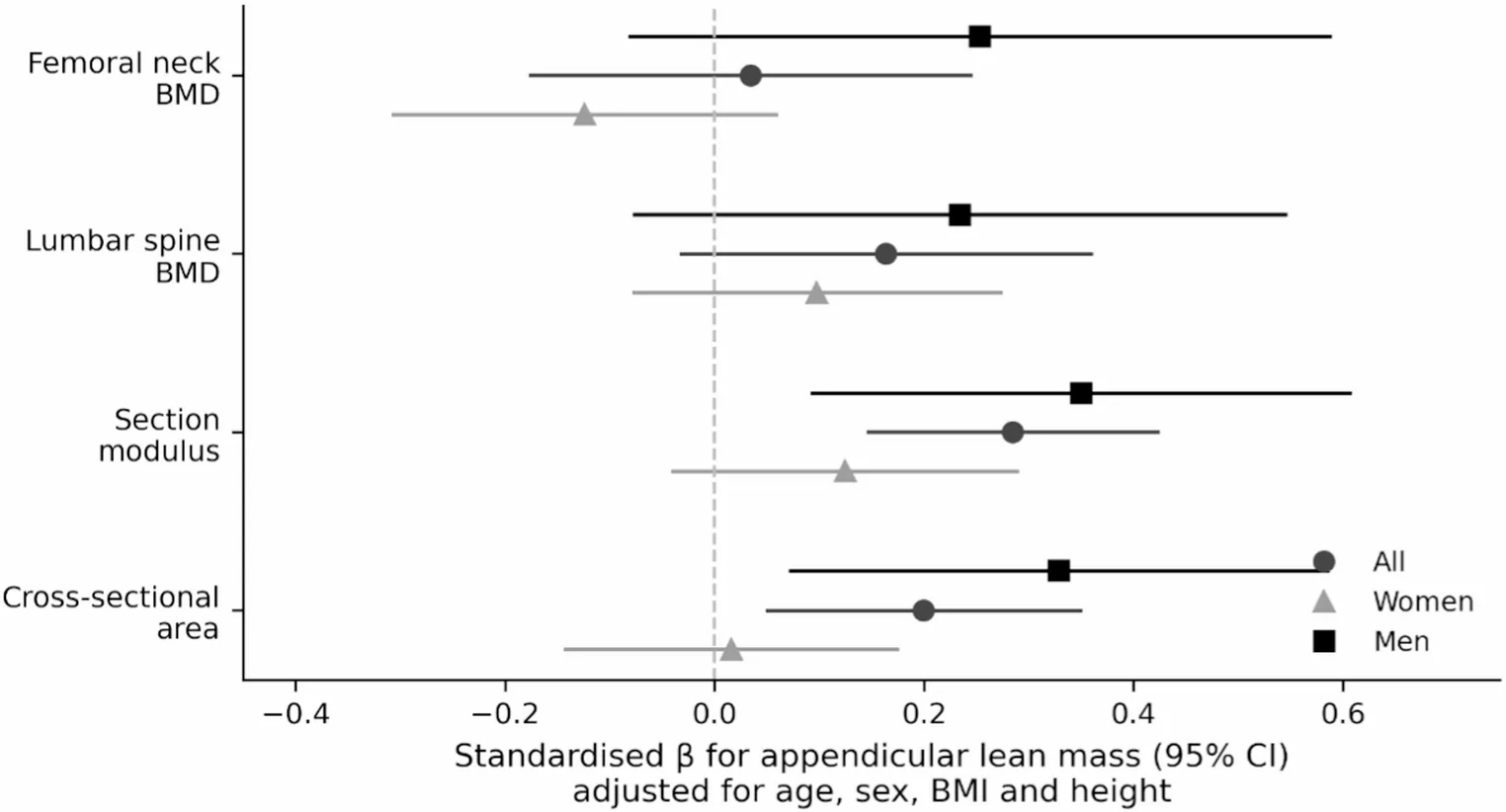
Standardised associations (95% CI) of appendicular lean mass with areal BMD and femoral geometry, overall and by sex. Given the significant sex interaction, the pooled (“All”) estimates are shown for completeness; the sex-specific estimates are more informative for interpretation

Appendicular lean mass was not statistically independently associated with femoral neck or lumbar spine areal BMD after adjustment for age, sex, BMI, and height (Table 5); progressive adjustment models are shown in Supplementary Table S6. However, the confidence intervals did not exclude small-to-moderate positive associations (upper limits 0.24 and 0.36 SD per SD of ALM, respectively). Lean mass was associated with femoral section modulus and cross-sectional area substantially more strongly in men than in women: on a common (globally standardised) scale the interaction was Δβ = 0.30 (95% CI 0.15 to 0.44, *p* < 0.001) for section modulus and Δβ = 0.24 (95% CI 0.08 to 0.39, *p* = 0.003) for cross-sectional area (Supplementary Table S7). In men the section modulus association persisted after adjustment for weight and height (standardised β = 0.31, 95% CI 0.06 to 0.57), in an allometric log–log model (elasticity 0.72, 95% CI 0.27 to 1.18), under heteroscedasticity-robust standard errors (*p* = 0.010), and after removal of influential observations (β = 0.29, *p* = 0.029) (Supplementary Table S8, Supplementary Figure S1). A within-subject contrast confirmed that lean mass was associated with femoral section modulus more strongly than with femoral neck areal BMD (difference 0.20 SD per SD of lean mass, bootstrap 95% CI 0.02 to 0.38; *p* = 0.026). Grip strength and SPPB were not independently associated with any bone outcome. Because the sex interaction was not an a priori hypothesis, the sex-specific pattern should be regarded as exploratory and requiring external replication (Fig. 3, Table 6).

**Table 5:** Variables independently associated with prevalent osteoporosis (logistic regression, n = 646)

Model	Variable	n	OR (95% CI)	*p*
Primary: age + male + BMI	Age (per year)	646	1.07 (1.03–1.11)	< 0.001
Primary: age + male + BMI	Male sex	646	0.35 (0.20–0.61)	< 0.001
Primary: age + male + BMI	Body mass index (per kg/m^2^)	646	0.87 (0.82–0.91)	< 0.001
Sensitivity: + FNIH confirmed sarcopenia	Confirmed sarcopenia (FNIH)	627	0.53 (0.15–1.90)	0.329
Sensitivity: + 25(OH)D	25(OH)D	463	1.00 (0.99–1.01)	0.768
Sensitivity: + PTH	PTH	355	1.00 (1.00–1.01)	0.593

**Table 6:** Standardised associations of appendicular lean mass with bone outcomes, overall and by sex (adjusted for age, sex where applicable, BMI and height)

Bone outcome	All β (95% CI)	Women β (95% CI)	Men β (95% CI)	p-int
Femoral neck BMD	n=529; 0.03 (− 0.18, 0.24)	n=375; -0.12 (− 0.31, 0.06)	n=154; 0.25 (− 0.08, 0.59)	0.17
Lumbar spine BMD	n=599; 0.16 (− 0.03, 0.36)	n=427; 0.10 (− 0.08, 0.27)	n=172; 0.23 (− 0.08, 0.54)	0.834
Section modulus	n=647; 0.28 (0.15, 0.42)	n=464; 0.12 (− 0.04, 0.29)	n=183; 0.35 (0.09, 0.61)	< 0.001
Cross-sectional area	n=647; 0.20 (0.05, 0.35)	n=464; 0.02 (− 0.14, 0.17)	n=183; 0.33 (0.07, 0.58)	0.002

Standardised β with 95% CI, standardised within each sex; p-int., p value for the appendicular lean mass × sex interaction estimated on globally standardised variables (Supplementary Table S7). Sensitivity analyses are given in the Supplementary material

## Discussion

In this community-dwelling cohort of older adults, the prevalence of low muscle mass varied more than fourfold, and that of confirmed sarcopenia threefold, depending only on how muscle mass was scaled to body size. Against that background, osteoporosis and sarcopenia showed no statistically significant excess co-occurrence. The overlap between osteoporosis and confirmed sarcopenia was not greater than expected under statistical independence for ALM/height2 or FNIH definitions, and was only slightly above expected for the absolute-ALM definition, with no statistically significant association. For probable sarcopenia, the largest and most precise analysis estimated an association close to unity and met the broad equivalence criterion, although not narrower equivalence criteria. These findings therefore provide no evidence for preferential co-segregation of low bone mass and sarcopenia in this community-based cohort, but larger studies are required to establish equivalence for confirmed sarcopenia. They do not prove that the two conditions are statistically or biologically independent. Shared genetic, endocrine, inflammatory, nutritional, and mechanical pathways may exist even without excess cross-sectional co-occurrence.

The dependence of prevalence on definition is itself an important result. In a cohort with a mean BMI of 29.6 kg/m^2^, the choice between height-squared normalisation and BMI adjustment changed the prevalence of low muscle mass from 3.7 to 15.7%. This is not merely a sensitivity analysis: it implies that a substantial part of the variation in reported osteosarcopenia prevalence across studies may reflect differences in operationalisation rather than in the underlying populations [8], and it cautions against pooling prevalence estimates derived under different muscle-mass metrics.

The secondary analysis provides a potential structural context for the low co-occurrence. Appendicular lean mass showed no statistically independent association with areal BMD after adjustment for body size, but was associated with femoral geometry, and a within-subject contrast confirmed that the geometric association was the stronger of the two. The two axes on which osteosarcopenia is defined, the areal BMD T-score and DXA muscle indices, may therefore capture partly distinct constructs, which is compatible with the observed low degree of co-occurrence. Consistent with the mechanostat model, the muscle–bone signal was structural rather than densitometric [14, 15, 19]. The association was substantially stronger in men than in women, indicating a sex-specific expression that requires confirmation in larger samples of older men; the interaction was robust to influential observations and to robust standard errors, but was based on 183 men. Because the association was observed for lean mass rather than grip strength, and because lean mass also reflects body size, nutrition, and shared developmental factors, a purely mechanical interpretation is not warranted [4].

The association of older age, female sex and lower BMI with osteoporosis is expected. Mean 25-hydroxyvitamin D was approximately 50 nmol/L and was not independently associated with prevalent osteoporosis in this sample. The high mean BMI (29.6 kg/m^2^) means the cohort is most informative for an overweight older population; leaner populations, in which ALM/height^2^ and ALM/BMI classifications diverge differently, may behave otherwise.

Our findings sit alongside a literature that has generally reported positive associations between sarcopenia and low BMD in clinical or fracture populations [5–7, 20]. The community setting, the use of multiple definitions and the explicit test against chance expectation distinguish the present analysis and support a more cautious reading of osteosarcopenia as, in part, the coincidence of two prevalent age-related conditions. If confirmed in larger community-based cohorts, this would suggest that part of the observed osteosarcopenia prevalence reflects the expected overlap of two common age-related conditions; however, preferential co-segregation of confirmed sarcopenia could not be excluded with adequate precision in the present study.

Recent studies further illustrate the heterogeneity of osteosarcopenia phenotypes and the potential clinical importance of muscle strength, body composition, and bone-muscle interactions for adverse outcomes and frailty [21, 22].

## Strengths and Limitations

Strengths include a community-dwelling sample assessed on a single densitometer, the use of three sarcopenia definitions with a formal test against chance and an equivalence analysis, and a validated linkage that did not rely on patient identifiers. Limitations include the cross-sectional, single-centre design; the small number of osteosarcopenia cases, which leaves the confirmed-definition analyses underpowered even though the result is consistent across definitions; the model-based nature of the hip structural analysis indices, which are estimates of geometry rather than direct measures of strength, and the absence of site-specific precision data for them; the possibility that lumbar spine BMD is artefactually raised by degenerative change at this age; and the modest number of men, so that the sex-specific muscle to geometry association requires confirmation. The structural explanation for the low co-occurrence is a hypothesis generated by cross-sectional data and would need mediation or longitudinal analysis to be established.

## Conclusion

In this community-dwelling cohort we found no evidence that osteoporosis and sarcopenia co-occurred more frequently than expected under statistical independence, across several operational definitions of sarcopenia; estimates for confirmed sarcopenia were imprecise because of the small number of co-occurring cases. The prevalence of low muscle mass and of osteosarcopenia depended strongly on the muscle-mass metric. The muscle–bone association was more apparent for femoral geometry than areal BMD and was observed predominantly in men. These findings do not support assuming that low bone mass and low muscle mass identify the same individuals, but they concern statistical co-occurrence and do not establish biological independence.

## Electronic Supplementary Material

Below is the link to the electronic supplementary material.

Supplementary Material 1
