# Supplementary material for: Osteosarcopenia: More Than the Sum of Its Parts? A Community-Based Study Across Sarcopenia Definitions
Source: Calcif Tissue Int. 2026 Oct 1;117(1):153. doi: 10.1007/s00223-026-01611-w (PMC13627189; doi:10.1007/s00223-026-01611-w)
Supplement: Supplementary file 1 [file 223_2026_1611_MOESM1_ESM.docx]

Supplementary materials

Analytic sample derivation. The source analytic dataset contained 699 participants. After excluding 42 participants aged under 65 years and one with missing age, 656 remained; seven had neither valid bone measurements nor hip structural analysis, leaving 649 participants in the main analytic cohort. Valid osteoporosis classification was available for 646 participants and hip structural analysis for 647 participants.

Supplementary analyses for: Osteosarcopenia: More Than the Sum of Its Parts? A Community-Based Study Across Sarcopenia Definitions.

All supplementary tables were generated from the single de-identified analytic dataset used in the main analysis bundle.

## Supplementary Table S1a. Missing data for participant and muscle variables

| **Variable** | **Available** | **Missing** | **Missing %** | **Missing women** | **Missing men** | **Missing OP+** | **Missing OP-** |
| --- | --- | --- | --- | --- | --- | --- | --- |
| Age | 656 | 0 | 0.0 | 0 | 0 | 0 | 0 |
| Sex | 656 | 0 | 0.0 | 0 | 0 | 0 | 0 |
| BMI | 655 | 1 | 0.2 | 1 | 0 | 0 | 0 |
| Height | 655 | 1 | 0.2 | 1 | 0 | 0 | 0 |
| Weight | 655 | 1 | 0.2 | 1 | 0 | 0 | 0 |
| ALM | 655 | 1 | 0.2 | 1 | 0 | 0 | 0 |
| ALM/height2 | 655 | 1 | 0.2 | 1 | 0 | 0 | 0 |
| ALM/BMI | 655 | 1 | 0.2 | 1 | 0 | 0 | 0 |
| Grip | 637 | 19 | 2.9 | 16 | 3 | 3 | 16 |
| Low grip | 637 | 19 | 2.9 | 16 | 3 | 3 | 16 |
| SPPB | 584 | 72 | 11.0 | 55 | 17 | 13 | 52 |

*OP, osteoporosis.*

## Supplementary Table S1b. Missing data for bone, laboratory and classification variables

| **Variable** | **Available** | **Missing** | **Missing %** | **Missing women** | **Missing men** | **Missing OP+** | **Missing OP-** |
| --- | --- | --- | --- | --- | --- | --- | --- |
| Lowest T-score | 646 | 10 | 1.5 | 9 | 1 | 0 | 0 |
| Any-site OP | 646 | 10 | 1.5 | 9 | 1 | 0 | 0 |
| Neck T-score | 529 | 127 | 19.4 | 97 | 30 | 16 | 101 |
| Total hip T-score | 529 | 127 | 19.4 | 97 | 30 | 16 | 101 |
| L1-L4 T-score | 599 | 57 | 8.7 | 45 | 12 | 10 | 37 |
| Neck BMD | 529 | 127 | 19.4 | 97 | 30 | 16 | 101 |
| L1-L4 BMD | 599 | 57 | 8.7 | 45 | 12 | 10 | 37 |
| Section modulus | 647 | 9 | 1.4 | 8 | 1 | 0 | 2 |
| CSA | 647 | 9 | 1.4 | 8 | 1 | 0 | 2 |
| 25(OH)D | 467 | 189 | 28.8 | 137 | 52 | 32 | 151 |
| PTH | 359 | 297 | 45.3 | 212 | 85 | 50 | 241 |
| Low mass ALM/ht2 | 655 | 1 | 0.2 | 1 | 0 | 0 | 0 |
| Low mass abs ALM | 655 | 1 | 0.2 | 1 | 0 | 0 | 0 |
| Low mass FNIH | 655 | 1 | 0.2 | 1 | 0 | 0 | 0 |
| Probable sarcopenia | 637 | 19 | 2.9 | 16 | 3 | 3 | 16 |
| Confirmed ALM/ht2 | 636 | 20 | 3.0 | 17 | 3 | 3 | 16 |
| Confirmed abs ALM | 636 | 20 | 3.0 | 17 | 3 | 3 | 16 |
| Confirmed FNIH | 636 | 20 | 3.0 | 17 | 3 | 3 | 16 |

*OP, osteoporosis.*

## Supplementary Table S2. Sex-stratified co-occurrence of osteoporosis and sarcopenia

| **Definition** | **Stratum** | **OP+/S+** | **OP+/S-** | **OP-/S+** | **OP-/S-** | **N** | **Observed %** | **Expected %** | **Odds ratio** |
| --- | --- | --- | --- | --- | --- | --- | --- | --- | --- |
| Confirmed ALM/ht2 | All | 1 | 110 | 7 | 509 | 627 | 0.16 | 0.23 | 0.66 |
| Confirmed ALM/ht2 | Women | 0 | 92 | 2 | 353 | 447 | 0.00 | 0.09 |  |
| Confirmed ALM/ht2 | Men | 1 | 18 | 5 | 156 | 180 | 0.56 | 0.35 | 1.73 |
| Confirmed abs ALM | All | 4 | 107 | 12 | 504 | 627 | 0.64 | 0.45 | 1.57 |
| Confirmed abs ALM | Women | 3 | 89 | 6 | 349 | 447 | 0.67 | 0.41 | 1.96 |
| Confirmed abs ALM | Men | 1 | 18 | 6 | 155 | 180 | 0.56 | 0.41 | 1.44 |
| Confirmed FNIH | All | 3 | 108 | 23 | 493 | 627 | 0.48 | 0.73 | 0.60 |
| Confirmed FNIH | Women | 2 | 90 | 14 | 341 | 447 | 0.45 | 0.74 | 0.54 |
| Confirmed FNIH | Men | 1 | 18 | 9 | 152 | 180 | 0.56 | 0.59 | 0.94 |
| Probable | All | 13 | 98 | 67 | 449 | 627 | 2.07 | 2.26 | 0.89 |
| Probable | Women | 11 | 81 | 49 | 306 | 447 | 2.46 | 2.76 | 0.85 |
| Probable | Men | 2 | 17 | 18 | 143 | 180 | 1.11 | 1.17 | 0.93 |

*OP, osteoporosis; S, sarcopenia.*

## Supplementary Table S3. TOST equivalence sensitivity for probable sarcopenia

| **Lower bound** | **Upper bound** | **Odds ratio** | **90% CI low** | **90% CI high** | **TOST p-value** | **Equivalent** |
| --- | --- | --- | --- | --- | --- | --- |
| 0.500 | 2.000 | 0.889 | 0.523 | 1.512 | 0.037 | Yes |
| 0.667 | 1.500 | 0.889 | 0.523 | 1.512 | 0.186 | No |
| 0.750 | 1.333 | 0.889 | 0.523 | 1.512 | 0.299 | No |

*TOST, two one-sided tests.*

## Supplementary Table S4. Osteoporosis-site sensitivity analyses

| **Definition** | **Site/stratum** | **OP+/S+** | **OP+/S-** | **OP-/S+** | **OP-/S-** | **N** | **Observed %** | **Expected %** | **Odds ratio** |
| --- | --- | --- | --- | --- | --- | --- | --- | --- | --- |
| Confirmed ALM/ht2 | Neck | 1 | 59 | 4 | 449 | 513 | 0.19 | 0.11 | 1.90 |
| Confirmed abs ALM | Neck | 3 | 57 | 9 | 444 | 513 | 0.58 | 0.27 | 2.60 |
| Confirmed FNIH | Neck | 2 | 58 | 18 | 435 | 513 | 0.39 | 0.46 | 0.83 |
| Probable | Neck | 6 | 54 | 55 | 398 | 513 | 1.17 | 1.39 | 0.80 |
| Confirmed ALM/ht2 | Total hip | 0 | 30 | 5 | 478 | 513 | 0.00 | 0.06 |  |
| Confirmed abs ALM | Total hip | 2 | 28 | 10 | 473 | 513 | 0.39 | 0.14 | 3.38 |
| Confirmed FNIH | Total hip | 1 | 29 | 19 | 464 | 513 | 0.19 | 0.23 | 0.84 |
| Probable | Total hip | 3 | 27 | 58 | 425 | 513 | 0.58 | 0.70 | 0.81 |
| Confirmed ALM/ht2 | Hip-only | 1 | 62 | 4 | 446 | 513 | 0.19 | 0.12 | 1.80 |
| Confirmed abs ALM | Hip-only | 3 | 60 | 9 | 441 | 513 | 0.58 | 0.29 | 2.45 |
| Confirmed FNIH | Hip-only | 2 | 61 | 18 | 432 | 513 | 0.39 | 0.48 | 0.79 |
| Probable | Hip-only | 6 | 57 | 55 | 395 | 513 | 1.17 | 1.46 | 0.76 |
| Confirmed ALM/ht2 | Lumbar spine | 0 | 61 | 7 | 514 | 582 | 0.00 | 0.13 |  |
| Confirmed abs ALM | Lumbar spine | 1 | 60 | 14 | 507 | 582 | 0.17 | 0.27 | 0.60 |
| Confirmed FNIH | Lumbar spine | 1 | 60 | 25 | 496 | 582 | 0.17 | 0.47 | 0.33 |
| Probable | Lumbar spine | 8 | 53 | 67 | 454 | 582 | 1.37 | 1.35 | 1.02 |
| Confirmed ALM/ht2 | Any site | 1 | 110 | 7 | 509 | 627 | 0.16 | 0.23 | 0.66 |
| Confirmed abs ALM | Any site | 4 | 107 | 12 | 504 | 627 | 0.64 | 0.45 | 1.57 |
| Confirmed FNIH | Any site | 3 | 108 | 23 | 493 | 627 | 0.48 | 0.73 | 0.60 |
| Probable | Any site | 13 | 98 | 67 | 449 | 627 | 2.07 | 2.26 | 0.89 |

*OP, osteoporosis; S, sarcopenia. Site/stratum identifies the osteoporosis definition.*

## Supplementary Table S5. Logistic models for osteoporosis by sarcopenia definition

| **Definition** | **Model** | **N** | **OP events** | **Odds ratio** | **95% CI low** | **95% CI high** | **p value** |
| --- | --- | --- | --- | --- | --- | --- | --- |
| Probable | crude | 627 | 111 | 0.889 | 0.472 | 1.674 | 0.716 |
| Probable | age + sex | 627 | 111 | 0.645 | 0.332 | 1.253 | 0.195 |
| Probable | age + sex + BMI | 627 | 111 | 0.593 | 0.299 | 1.177 | 0.135 |
| Confirmed ALM/ht2 | crude | 627 | 111 | 0.661 | 0.081 | 5.427 | 0.700 |
| Confirmed ALM/ht2 | age + sex | 627 | 111 | 0.614 | 0.071 | 5.332 | 0.658 |
| Confirmed ALM/ht2 | age + sex + BMI | 627 | 111 | 0.332 | 0.038 | 2.912 | 0.320 |
| Confirmed abs ALM | crude | 627 | 111 | 1.570 | 0.497 | 4.962 | 0.442 |
| Confirmed abs ALM | age + sex | 627 | 111 | 1.154 | 0.346 | 3.852 | 0.815 |
| Confirmed abs ALM | age + sex + BMI | 627 | 111 | 0.730 | 0.212 | 2.515 | 0.618 |
| Confirmed FNIH | crude | 627 | 111 | 0.595 | 0.176 | 2.019 | 0.405 |
| Confirmed FNIH | age + sex | 627 | 111 | 0.510 | 0.147 | 1.767 | 0.288 |
| Confirmed FNIH | age + sex + BMI | 627 | 111 | 0.527 | 0.146 | 1.905 | 0.329 |

*Odds ratios are for the sarcopenia term in each model.*

## Supplementary Table S6. Progressive ALM-bone models: Neck BMD

| **Model** | **N** | **Std beta** | **95% CI low** | **95% CI high** | **p value** |
| --- | --- | --- | --- | --- | --- |
| unadjusted | 529 | 0.515 | 0.442 | 0.589 | 0.000 |
| age + sex | 529 | 0.459 | 0.352 | 0.567 | 0.000 |
| age + sex + height | 529 | 0.411 | 0.287 | 0.535 | 0.000 |
| age + sex + height + weight | 529 | -0.010 | -0.225 | 0.204 | 0.925 |
| age + sex + BMI + height | 529 | 0.034 | -0.176 | 0.244 | 0.750 |
| allometric log-log | 529 | -0.117 | -0.279 | 0.045 | 0.156 |

*ALM, appendicular lean mass; Std beta, standardised beta.*

## Supplementary Table S6. Progressive ALM-bone models: L1-L4 BMD

| **Model** | **N** | **Std beta** | **95% CI low** | **95% CI high** | **p value** |
| --- | --- | --- | --- | --- | --- |
| unadjusted | 599 | 0.525 | 0.456 | 0.593 | 0.000 |
| age + sex | 599 | 0.480 | 0.380 | 0.580 | 0.000 |
| age + sex + height | 599 | 0.507 | 0.392 | 0.621 | 0.000 |
| age + sex + height + weight | 599 | 0.129 | -0.072 | 0.329 | 0.208 |
| age + sex + BMI + height | 599 | 0.163 | -0.032 | 0.359 | 0.101 |
| allometric log-log | 599 | 0.026 | -0.147 | 0.199 | 0.766 |

*ALM, appendicular lean mass; Std beta, standardised beta.*

## Supplementary Table S6. Progressive ALM-bone models: Section modulus

| **Model** | **N** | **Std beta** | **95% CI low** | **95% CI high** | **p value** |
| --- | --- | --- | --- | --- | --- |
| unadjusted | 647 | 0.726 | 0.672 | 0.779 | 0.000 |
| age + sex | 647 | 0.466 | 0.393 | 0.538 | 0.000 |
| age + sex + height | 647 | 0.329 | 0.248 | 0.410 | 0.000 |
| age + sex + height + weight | 647 | 0.251 | 0.109 | 0.392 | 0.001 |
| age + sex + BMI + height | 647 | 0.285 | 0.146 | 0.423 | 0.000 |
| allometric log-log | 647 | 0.253 | 0.029 | 0.476 | 0.027 |

*ALM, appendicular lean mass; Std beta, standardised beta.*

## Supplementary Table S6. Progressive ALM-bone models: CSA

| **Model** | **N** | **Std beta** | **95% CI low** | **95% CI high** | **p value** |
| --- | --- | --- | --- | --- | --- |
| unadjusted | 647 | 0.704 | 0.649 | 0.759 | 0.000 |
| age + sex | 647 | 0.536 | 0.458 | 0.614 | 0.000 |
| age + sex + height | 647 | 0.412 | 0.324 | 0.501 | 0.000 |
| age + sex + height + weight | 647 | 0.164 | 0.012 | 0.317 | 0.035 |
| age + sex + BMI + height | 647 | 0.200 | 0.050 | 0.349 | 0.009 |
| allometric log-log | 647 | 0.036 | -0.115 | 0.188 | 0.638 |

*ALM, appendicular lean mass; Std beta, standardised beta.*

## Supplementary Table S7a. ALM by sex interaction models: raw scale

| **Outcome** | **N** | **Raw interaction** | **SE** | **Raw 95% CI low** | **Raw 95% CI high** | **Raw p** |
| --- | --- | --- | --- | --- | --- | --- |
| Section modulus | 647 | 15.120 | 3.720 | 7.830 | 22.411 | 0.000 |
| CSA | 647 | 1.758 | 0.580 | 0.621 | 2.894 | 0.002 |

*ALM, appendicular lean mass.*

## Supplementary Table S7b. ALM by sex interaction models: globally standardised scale

| **Outcome** | **N** | **Std delta beta** | **Std 95% CI low** | **Std 95% CI high** | **Std p** |
| --- | --- | --- | --- | --- | --- |
| Section modulus | 647 | 0.295 | 0.153 | 0.437 | 0.000 |
| CSA | 647 | 0.239 | 0.084 | 0.393 | 0.002 |

*Std, globally standardised.*

## Supplementary Table S8. Sensitivity analyses in men

| **Outcome** | **Model** | **N** | **Estimate** | **95% CI low** | **95% CI high** | **p value** |
| --- | --- | --- | --- | --- | --- | --- |
| Section modulus | age + weight + height | 183 | 0.312 | 0.055 | 0.568 | 0.017 |
| Section modulus | allometric log-log | 183 | 0.724 | 0.271 | 1.177 | 0.002 |
| Section modulus | HC3 robust SE | 183 | 21.334 | 5.020 | 37.647 | 0.010 |
| Section modulus | exclude Cook's D > 0.0219 | 174 | 0.291 |  |  | 0.029 |
| Section modulus | VIF asm in BMI + height model | 183 | 4.602 |  |  |  |
| CSA | age + weight + height | 183 | 0.293 | 0.038 | 0.549 | 0.025 |
| CSA | allometric log-log | 183 | 0.327 | 0.014 | 0.640 | 0.041 |
| CSA | HC3 robust SE | 183 | 2.916 | 0.456 | 5.377 | 0.020 |
| CSA | exclude Cook's D > 0.0219 | 175 | 0.135 |  |  | 0.350 |
| CSA | VIF asm in BMI + height model | 183 | 4.602 |  |  |  |

*VIF, variance inflation factor; HC3, heteroscedasticity-consistent standard error.*

## Supplementary Table S9. Within-subject contrast

| **Contrast** | **N** | **Delta** | **95% CI low** | **95% CI high** | **p value** | **Resamples** | **Seed** |
| --- | --- | --- | --- | --- | --- | --- | --- |
| Section modulus association minus femoral neck BMD association | 529 | 0.203 | 0.022 | 0.375 | 0.026 | 2000 | 42 |

*Delta is the difference in standardised association with section modulus versus femoral neck BMD.*

## Supplementary Table S10. Severe sarcopenia prevalence

| **Group** | **Measure** | **n** | **N** | **%** | **95% CI low** | **95% CI high** |
| --- | --- | --- | --- | --- | --- | --- |
| Severe sarcopenia | Severe sarcopenia (ALM/height2; confirmed + SPPB <= 8) | 3 | 575 | 0.5 | 0.2 | 1.5 |
| Osteosarcopenia severe | Osteoporosis + severe sarcopenia (ALM/height2) | 1 | 572 | 0.2 | 0.0 | 1.0 |
| Severe sarcopenia | Severe sarcopenia (absolute ALM; confirmed + SPPB <= 8) | 8 | 575 | 1.4 | 0.7 | 2.7 |
| Osteosarcopenia severe | Osteoporosis + severe sarcopenia (absolute ALM) | 3 | 572 | 0.5 | 0.2 | 1.5 |
| Severe sarcopenia | Severe sarcopenia (FNIH; confirmed + SPPB <= 8) | 15 | 575 | 2.6 | 1.6 | 4.3 |
| Osteosarcopenia severe | Osteoporosis + severe sarcopenia (FNIH) | 2 | 572 | 0.3 | 0.1 | 1.3 |

## Supplementary Figure S1. Appendicular lean mass and femoral section modulus by sex
